# Anti-Müllerian Hormone and Its Clinical Use in Pediatrics with Special Emphasis on Disorders of Sex Development

**DOI:** 10.1155/2013/198698

**Published:** 2013-12-03

**Authors:** Marie Lindhardt Johansen, Casper P. Hagen, Trine Holm Johannsen, Katharina M. Main, Jean-Yves Picard, Anne Jørgensen, Ewa Rajpert-De Meyts, Anders Juul

**Affiliations:** ^1^Department of Growth and Reproduction, GR, 5064 Rigshospitalet, Faculty of Medical and Health Sciences, University of Copenhagen, Blegdamsvej 9, 2100 Copenhagen, Denmark; ^2^INSERM U782 Research Unit and Paris Sud University, 92140 Clamart, France

## Abstract

Using measurements of circulating anti-Müllerian hormone (AMH) in diagnosing and managing reproductive disorders in pediatric patients requires thorough knowledge on normative values according to age and gender. We provide age- and sex-specific reference ranges for the Immunotech assay and conversion factors for the DSL and Generation II assays. With this tool in hand, the pediatrician can use serum concentrations of AMH when determining the presence of testicular tissue in patients with bilaterally absent testes or more severe Disorders of Sex Development (DSD). Furthermore, AMH can be used as a marker of premature ovarian insufficiency (POI) in both Turner Syndrome patients and in girls with cancer after treatment with alkylating gonadotoxic agents. Lastly, its usefulness has been proposed in the diagnosis of polycystic ovarian syndrome (PCOS) and ovarian granulosa cell tumors and in the evaluation of patients with hypogonadotropic hypogonadism.

## 1. Introduction

Anti-Müllerian hormone (AMH), also known as Müllerian inhibiting substance, is essential for the involution of the Müllerian ducts (the anlagen of the internal female genitalia) in the male fetus [1–3]. Male sex differentiation is completely dependent on the normal development of testes that produce ample amounts of testosterone and AMH. The two hormones, produced by Leydig cells and Sertoli cells, respectively, represent two distinct pathways in male sex differentiation. Testosterone is responsible for the differentiation of the Wolffian ducts, the urogenital sinus, and the external genitalia. By contrast, AMH does not have any known function in female fetal organogenesis.

Determination of the serum AMH concentration is used in various ways in clinical pediatrics to determine the presence of testicular tissue in patients with cryptorchidism, suspected anorchia, or more severe Disorders of Sex Development (DSD). Also, AMH may be used as a marker of premature ovarian insufficiency (POI). It has been proposed as a marker in polycystic ovarian syndrome (PCOS) [4], as a tumor load marker in ovarian granulosa cell tumors [5] and, lastly, in hypogonadotropic hypogonadism [6].

This paper gives a brief overview of the physiology of AMH and seeks to give clinicians a tool when interpreting results from different assays. The aim is to simplify the use of AMH in clinical pediatrics.

## 2. Expression and Regulation of AMH

**Figure 1:**
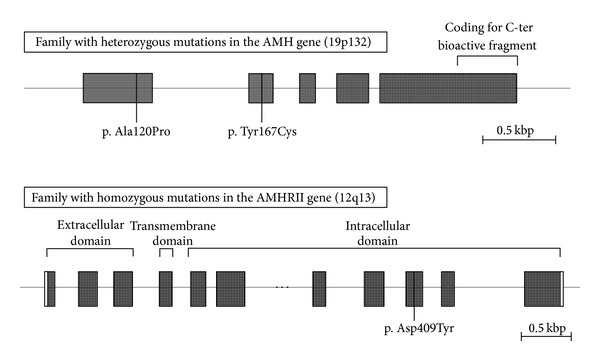
Mutations of the *AMH* gene (Case 1) and the *AMH-RII* gene (Case 2) in two families with boys presenting with PMDS. Two missense mutations in exons 1 and 2 (substitution of alanine by proline and of tyrosine by cysteine, resp.) were found in the *AMH* gene in two brothers presenting with AMH-negative PMDS. A homozygous missense mutation in exon 9 (substitution of aspartic acid by tyrosine) in the *AMH-RII* gene was found in two brothers presenting with AMH-positive PMDS.

AMH is produced by Sertoli and granulosa cells in the male and female, respectively. AMH is a member of the TGF-*β* family and is encoded by the *AMH* gene, which contains 5 exons [7] and is located on chromosome 19 p13.3 [8] (see Figure 1 for an iconography of the gene). The hormone binds to the specific AMH type II receptor (AMH-RII), which is encoded by a gene found on chromosome 12 p13 (see Figure 1 for an iconography of the gene) [9]. The receptor is a single transmembrane protein with serine-threonine kinase activity, and it is present on the cell membrane of target organs (including the mesenchymal cells of the Müllerian ducts, the granulosa cells of the ovary, and the Sertoli and Leydig cells of the testis) [10].

Several genes play a role in the regulation of AMH production from Sertoli cells. The *SRY *gene, for example, is important for the activation of *SOX9*, which together with steroidogenic factor 1 (SF1) and DAX1 directly stimulates the expression of *AMH* in the fetal testes [14, 15].

## 3. Male and Female Serum AMH Reference Ranges

**Figure 2:**
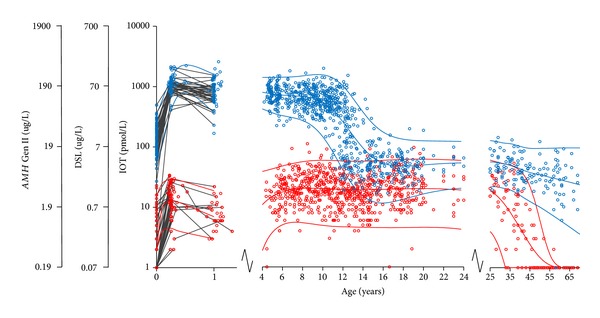
Serum AMH in 1953 healthy subjects (926 females and 1027 males) according to age. Females: red circles, males: blue circles. Longitudinal values during infancy are connected with grey lines. The red and blue curves represent the female and male reference ranges, respectively (median, ±2SD). The figure was redrawn from our previously published data using Immunotech Coulter enzyme immunometric assay in all subjects [11–13]. Please note the logarithmic *y*-axis. The *y*-axes for the DSL and Gen II assays were created using the following formulas: AMH (IOT) pmol/L = 2.0 × AMH (DSL) ug/L × 7.14 pmol/ug and AMH (IOT) pmol/L = 0.74 × AMH (Gen II) ug/L × 7.14 pmol/ug.

We have previously reported serum AMH reference ranges for males and females throughout the entire lifespan measured on the Immunotech Coulter assay [11, 12], (Figure 2). At birth, male cord blood has high levels of AMH (mean 148 pmol/L), whereas AMH is undetectable (54%) or very low (95% CI: <2–16) in cord sera from female infants. At three months of age, AMH levels increase markedly in both sexes, although the concentrations in females (mean 13 pmol/L) remain much lower compared to concentrations in male infants (mean 1047 pmol/L). During childhood, AMH levels are relatively stable in both sexes, boys having approximately 35 times higher levels than girls.

Until pubertal onset, AMH is consequently a sensitive and specific marker of Sertoli cell activity. With the onset of testosterone synthesis in male puberty, serum AMH levels decline rapidly (mean 50 pmol/L), which clearly overlap with the levels seen in healthy females (Figure 2).

## 4. The Secretion and Function of AMH

### 4.1. AMH in Males

**Figure 3:**
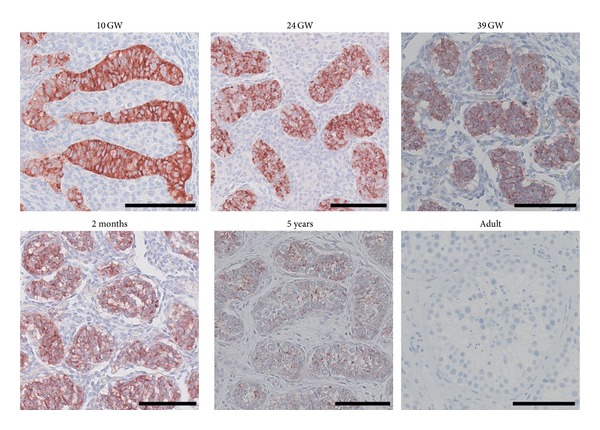
Immunohistochemical staining of AMH in testis tissue. GW: gestational week. Scale bar corresponds to 100 *μ*m.

In males, the Sertoli cells begin to secrete AMH during the 7th week of gestation.  Figure 3, modified from a series by Jørgensen et al. [16], clearly shows the high expression of AMH in the fetal testis and furthermore illustrates the reduction of AMH expression in Sertoli cells with increasing age and testosterone production which is also reflected in serum values [12, 17–19]. The adult male testis shows an absence of immunohistochemical staining for AMH. The origin of AMH in adult male serum is unknown, although testicular origin seems most likely despite the expression pattern seen in Figure 3. This is supported by visible AMH expression in slightly undifferentiated Sertoli cells that are occasionally seen in infertile men, often in Sertoli-cell-only tubules [20, 21].

The postnatal surge of AMH, which is seen in Figure 2, is most likely triggered by increasing concentrations of follicle-stimulating hormone (FSH) [22–24]. The pubertal decline is most likely caused by androgens after pubertal activation of the androgen receptor in the differentiated Sertoli cell [6, 17, 21, 25]. Inhibition probably also happens via the synergy with maturing germ cells rather than only through a direct inhibition of AMH transcription [26–28].

### 4.2. AMH in Females

In female fetuses, AMH is not present in ovarian tissues until the 36th week of gestation [21]. Female AMH is produced by ovarian granulosa cells of the preantral and early antral follicles [29–31]. The circulating levels of AMH in adult women reflect the number of remaining primordial follicles [32].

During childhood and adolescence, AMH fluctuations are minimal and each girl maintains her relative level during pubertal transition [33, 34]. The stable AMH levels during the extensive loss of primordial follicles in childhood are probably balanced on one hand by an increased recruitment rate of primordial follicles during childhood (supplying AMH-producing follicles) and on the other hand by FSH-induced follicle growth at the time of pubertal onset (more follicles grow beyond AMH producing stages) [35].

In female mice, AMH inhibits primordial follicle recruitment and growth, which probably plays an important role in the maintenance of the follicle pool [36]. AMH furthermore plays a role in the regulation of ovarian steroidogenesis by inhibiting aromatase activity [37] and decreasing intrafollicular estradiol concentrations [38, 39].

## 5. AMH Assays

At present, three different AMH assays exist: Diagnostic Systems Lab (DSL by Diagnostic Systems Laboratories, Webster, TX, USA), Immunotech (IOT by Beckman Coulter, Inc., Brea, CA, USA), and the new AMH Generation II assay (Gen II by Beckman Coulter, Inc., Brea, CA, USA). The Gen II assay combines antibodies from the DSL and calibration standards from the IOT [40, 41]. Until the Gen II assay is ubiquitously implemented, there is a need for assay-specific reference ranges for clinicians to use. Few comparative studies between AMH assays have been performed, but Gen II levels appear to correlate well with both DSL and IOT, although the new assay produces higher values [41–45].

Previously, we reported age- and sex-specific reference ranges [13]; this figure has now been modified to make it assay-specific too, see Figure 2. It is important to note that we have not performed comparative studies with the testing of identical samples on the three different assays. The figure is merely a translation between assays based on current literature.

In Figure 2, the samples have been run with the IOT assay (Immunotech, Marseilles, France) [11, 12]. We use a conversion factor of 2.0 between DSL and IOT, which has previously been suggested and is widely accepted [11, 46, 47]. The units used by IOT and DSL are pmol/L and ug/L, respectively; it is therefore necessary to account for the molecular weight of AMH (140 kDa = 140 g/mol = 7.14 pmol/ug). Consider

(1) AMH(IOT)pmol/L=2.0×AMH(DSL)ug/L ×7.14 pmol/ug.

The *y*-axis for Gen II has been created based on a conversion factor of 0.74 as reached by Li et al. [42], which, to our knowledge, is the only study to date that has run identical samples on both the IOT and Gen II assays. Consider

(2) AMH(IOT)pmol/L=0.74×AMH(Gen  II)ug/L ×7.14 pmol/ug.

The linearity of the relationship at higher values is questioned [42]. The clinical value of AMH measurements during childhood does, however, seem to be preserved despite the uncertainty of high-end values, as the value mostly lies in the distinguishing between low female and high male values in different clinical scenarios.

## 6. AMH and Its Clinical Use in Pediatric Patients

AMH has a wide clinical potential in pediatrics. Figure 2 provides a clinical tool where a specific AMH value can be interpreted in an age-, sex-, and assay-specific manner.

In the following section, we describe clinical conditions in which detectable versus undetectable AMH measurements are important for diagnosis.

### 6.1. Determination of the Presence of Testicular Tissue

When a child is born with ambiguous genitalia or bilaterally absent testes (as in Case 1), the determination of the presence or absence of testicular tissue is of utmost importance in terms of treatment and management options. A serum AMH concentration within the normal male reference range is highly indicative of testicular tissue [48–50]. In patients with ambiguous genitalia or cryptorchidism, a low or undetectable AMH concentration is conversely indicative of dysgenetic testicular tissue, anorchia (as seen in Case 1) [49–53] or ovarian tissue [48]. AMH can thus be used to distinguish between anorchia and cryptorchidism in patients without palpable testes.

In patients with ambiguous genitalia, an AMH value within the normal male reference range indicates intact Sertoli cell function. In the case of patients undergoing surgery, for example, a girl with an ovotestis, AMH can be used as a marker of testicular tissue before and after surgery [54].

*The Relevance of Human Chorionic Gonadotropin (hCG) Tests in Patients with Cryptorchidism*. hCG testing may not be necessary in determining the absence of testicular tissue; a simple AMH measurement has been suggested to have a higher predictive value than hCG stimulated testosterone levels (hCG stimulation may fail to increase serum testosterone in some patients with abdominal testes [48]), depending on whether or not hCG is given once or repetitively and on the patient's age [48, 50, 52].

In the case of a patient with undetectable levels of AMH and bilateral cryptorchidism, an hCG test should, however, be performed to asses Leydig cell function and to exclude the rare diagnosis of Persistent Müllerian Duct Syndrome [48, 55]. A lack of increased stimulated testosterone concludes the diagnosis of anorchia (see Case 1), whereas increased concentrations indicate the presence of testicular tissue, and that Persistent Müllerian Duct Syndrome (PMDS) should be considered (see below and Cases 2 and 3) [48].

AMH has furthermore been suggested as a marker used in the management of patients with an isolated microphallus or hypospadias to exclude the possibility of testicular dysgenesis. Few patients, however, have subnormal values indicating testicular dysgenesis as the underlying cause [50, 53, 56].

Hence, measuring serum AMH is mostly of great clinical value when dealing with patients with ambiguous genitalia or bilateral cryptorchidism.

### 6.2. Persistent Müllerian Duct Syndrome

Abnormal AMH secretion or action leads to the persistence of the Müllerian ducts, that is, uterus, Fallopian tubes, and the upper part of the vagina. This can stem from a mutation in the gene encoding AMH or the AMH type II receptor (AMH-RII), known as PMDS, or it can be a sign of testicular dysgenesis. In the latter case, the phenotype will also be affected by disordered androgen secretion leading to external sexual ambiguity.

Patients with PMDS are referred to as either AMH-negative or -positive (with or without detectable serum concentrations of AMH, resp.), which gives clues to the underlying genetic defect [57]. AMH-negative PMDS is indicative of *AMH* gene mutations (as in Case 2, gene mutation illustrated in Figure 1), whereas AMH-positive PMDS leads to a suspicion of mutations in the *AMH-RII* gene (as in Case 3, gene mutation illustrated in Figure 1). In exceptional cases, however, AMH-positive PMDS may be due to a mutation in the *AMH *gene, which affects AMH bioactivity rather than secretion [58].

A fairly large group of PMDS patients (about 13–15% as reported by Josso et al. [57, 59]) remain without a genetic diagnosis, that is, no mutations found in neither the *AMH* nor the *AMH-RII *genes.

PMDS patients are phenotypically divided into two groups independent of the underlying genetic defect: patients born with bilateral cryptorchidism and patients born with one descended testis that drags the ipsilateral Fallopian tube into the inguinal canal creating a hernia and a contralateral abdominal testis [57, 60].

### 6.3. Virilized Females

AMH concentrations also indicate whether the virilization of a girl originates from testicular tissue or a granulosa cell tumor (AMH concentrations in the male reference range) or from adrenal androgens, that is, in the case of congenital adrenal hyperplasia (AMH concentrations in the female reference range). Values within the normal female reference range do not exclude the presence of abnormal gonadal tissue, but undetectable levels exclude the presence of testicular tissue in mildly virilized females [55, 61].

In the following section, we will describe clinical conditions in which the relative AMH concentration is important.

### 6.4. Low Serum Concentrations of AMH

*Premature Ovarian Insufficiency*. In pediatrics, Turner Syndrome patients experiencing accelerated loss of ovarian follicles and cancer patients receiving alkylating gonadotoxic treatment represent the majority of cases of premature ovarian insufficiency (POI). Low or undetectable AMH seems to be an excellent marker of POI in these patients [62–64].

### 6.5. Normal Serum Concentrations of AMH

#### 6.5.1. Hypogonadotropic Hypogonadism

Hypogonadotropic hypogonadism (HH) includes an array of disorders all characterized by low or absent endogenous gonadotropins and consequently low or undetectable sex hormones in pediatric as well as adult patients.

In prepubertal and pubertal males with HH, AMH levels may be subnormal compared to the male reference range [65], whereas postpubertal levels may be high compared to normal male levels [66]. These phenomena could be explained by a lack of FSH stimulus prepubertally and a lack of intratesticular testosterone-mediated downregulation of AMH secretion during and after puberty, respectively [6, 22, 23, 67]. Pubertal and postpubertal levels may, however, also be low in patients with more severe HH. This is probably a consequence of the missing FSH stimulus and the subsequently smaller pool of Sertoli cells causing a deficient AMH secretion [68].

AMH has been proposed as a tool in differentiating between HH and constitutional pubertal delay but has not yet been proven clinically valuable [65], and some conclude that inhibin B seems to be the better marker of the two [68].

In females, AMH is low in the reference range due to the partial gonadotropin-dependent regulation of AMH [69]. Traditional endocrine evaluation (gonadotropins, inhibin B, and estradiol) remains as the preferred diagnostic tool for this condition, but AMH appears to be a promising marker of ovarian response in idiopathic HH patients with induced menstrual cycles [70]. This is important in fertility clinics but not as important in a pediatric setting.

Consequently, AMH is not yet of particular clinical use in pediatrics when suspecting HH in patients of either sex.

#### 6.5.2. Klinefelter Syndrome (47,XXY)

Klinefelter Syndrome (KS) is characterized by small testes, tall stature, and adult hypergonadotropic hypogonadism. Boys with KS have normal AMH concentrations until puberty [71–75]. After the expected pubertal decline, AMH values fall to subnormal concentrations [71, 72, 74, 76]. This may be explained by a progressive destruction of the testes seen in patients with 47,XXY [71]. AMH thus seems to be an excellent marker of testicular function in these patients.

### 6.6. High Serum Concentrations of AMH

#### 6.6.1. Granulosa Cell Tumors

Granulosa cell tumors are sex cord tumors that make up 2–5% of ovarian neoplasms [77–80]. Only a few percent, however, are seen prepubertally [81]. In prepubertal girls, granulosa cell tumors may present as precocious puberty [82, 83]. In 1992, AMH was first identified as a granulosa tumor marker [84]. Serum AMH has been shown to be a fairly reliable and specific marker of granulosa cell tumors and their activity [83, 85–90]. In advanced stages, however, the large tumors may gradually lose their AMH expression, rendering AMH as a less reliable marker in these more advanced cases [5, 82]. One study directly found that tumor size was inversely related to AMH expression [91].

In a clinical pediatric setting, a prepubertal girl presenting with masculine AMH concentrations should be thoroughly examined for the presence of testes (i.e., in the complete type of androgen insensitivity syndrome) or of ovarian pathology (i.e., a granulosa cell tumor). A granulosa cell tumor cannot, however, be excluded in a girl presenting with AMH values within the reference range.

AMH, together with inhibin, has furthermore been proposed as a residual and relapse tumor marker postsurgically [85].

#### 6.6.2. PCOS

Polycystic ovarian syndrome (PCOS) causes anovulatory infertility and hyperandrogenism along with elevated AMH in premenopausal women [4, 92–94]. AMH has not, however, been proven as a sensitive and specific diagnostic marker of neither PCOS nor of polycystic ovarian morphology [95]. Clear cutoffs do not exist in adolescence [96, 97], and several thresholds have been suggested in adults with varying degrees of sensitivity and specificity [4, 95, 98, 99].

Initially following menarche, anovulatory irregular menstrual bleedings are common and often resolve without diagnosis or treatment [100–102]. If an adolescent girl presents with a prolonged period of oligomenorrhea, an elevated serum AMH value may indicate underlying PCOS [92, 103, 104]. AMH is only mildly affected by the menstrual cycle and lowered by oral contraceptives [46, 105, 106], making it a useful marker for underlying pathology, namely, PCOS, in these patients when a high AMH concentration is found.

In conclusion, AMH is a very useful serum marker of gonadal differentiation and function in pediatric reproductive disorders. This paper has sought to simplify the correlation between the three AMH assays and review the usefulness in diagnosing and managing patients with various pediatric disorders of reproductive endocrinology.

## 7. Cases

### 7.1. Case 1: Boy Born with Anorchia

This boy was born with a hypoplastic scrotum, no palpable testes in the scrotum or inguinal canal, and an extreme micropenis. Laparoscopy at the age of 4 years was performed, and normal vasa deferentia were found along with normal testicular vessels. However, no testicles were located intra-abdominally. An hCG test at the age of 6 showed testosterone levels below 0.23 nmol/L. This concluded the diagnosis of anorchia, which was furthermore supported by undetectable AMH concentrations. Inhibin B was undetectable at all times. LH was immeasurable and FSH was between 1.37 and 1.63 U/L prior to puberty induction. Supplemental testosterone treatment was started at the age of 10.5 to induce puberty.

### 7.2. Case 2: AMH-Negative PMDS Patient

This patient was born at term with a left retractile testis and a right scrotal testis. At one month of age, a right-sided inguinal hernia appeared. The karyotype was that of a normal male, 46,XY. Inhibin B concentration was normal (402 pg/mL), and serum values of FSH, LH, and testosterone were all within the normal reference range for age. Herniotomy at the age of three months revealed an ovary-looking organ with a Fallopian tube attached bilaterally. Histology showed normal testicular tissue and orchidopexy was performed. Subsequently, laparoscopy was carried out and a uterus was found and removed including bilateral Fallopian tubes. Serum AMH was undetectable and, consequently, the *AMH* gene was sequenced. As illustrated in Figure 1, this resulted in the findings of two heterozygous missense mutations in exons 1 and 2 of *AMH*, respectively, that is, a substitution of the nonpolar alanine by the nonpolar proline (p.Ala120Pro) in exon 1 and a substitution of the polar tyrosine by the polar cysteine (p.Tyr167Cys) in exon 2.

One and a half year after the birth of this boy, a younger brother was born with a similar phenotype and the same *AMH* mutations were demonstrated.

### 7.3. Case 3: AMH-Positive PMDS Patient

This patient was born at term with nonpalpable bilateral cryptorchidism. The karyotype was that of a normal male, 46,XY. An hCG test at the age of 4.5 years resulted in rises in serum testosterone from <0.23 to 2.65 (at day 3) and 1.86 nmol/L (at day 4), respectively. Serum inhibin B was undetectable, and concentrations of FSH and LH levels were normal. Laparoscopy at the age of two years revealed the presence of intra-abdominal testes, a uterus, and bilateral Fallopian tubes; orchidopexy and hysterectomy were, thus, performed. Serum AMH was detectable in 5 of 5 samples varying from 17 to 75 pmol/L. Consequently, the *AMH-RII* gene was sequenced. As illustrated in Figure 1, a homozygous missense mutation in *AMH-RII* was confirmed, that is, a substitution of the polar aspartic acid by the polar tyrosine (p.Asp490Tyr) in exon 9.

Three years after the birth of this boy, a younger brother was born with a similar phenotype and the same *AMH-RII* mutation was demonstrated.
